# Biochar Optimizes Wheat Quality, Yield, and Nitrogen Acquisition in Low Fertile Calcareous Soil Treated With Organic and Mineral Nitrogen Fertilizers

**DOI:** 10.3389/fpls.2022.879788

**Published:** 2022-05-03

**Authors:** Mushtaq Ahmad Khan, Abdul Basir, Shah Fahad, Muhammad Adnan, Muhammad Hamzah Saleem, Anas Iqbal, Amanullah, Asma A. Al-Huqail, Areej A. Alosaimi, Shah Saud, Ke Liu, Matthew Tom Harrison, Taufiq Nawaz

**Affiliations:** ^1^Department of Agriculture, University of Swabi, Swabi, Pakistan; ^2^Hainan Key Laboratory for Sustainable Utilization of Tropical Bioresource, College of Tropical Crops, Hainan University, Haikou, China; ^3^Department of Agronomy, Faculty of Agricultural Sciences, The University of Haripur, Haripur, Pakistan; ^4^College of Plant Science and Technology, Huazhong Agricultural University, Wuhan, China; ^5^College of Life Science and Technology, Guangxi University, Nanning, China; ^6^Department of Agronomy, The University of Agriculture Peshawar, Peshawar, Pakistan; ^7^Chair of Climate Change, Environmental Development and Vegetation Cover, Department of Botany and Microbiology, College of Science, King Saud University, Riyadh, Saudi Arabia; ^8^Department of Biology, College of Science, Imam Abdulrahman Al Faisal University, Dammam, Saudi Arabia; ^9^College of Life Science, Linyi University, Linyi, China; ^10^Hubei Collaborative Innovation Center for Grain Industry/Agriculture College, Yangtze University, Jingzhou, China; ^11^Tasmanian Institute of Agriculture, University of Tasmania, Burnie, TAS, Australia; ^12^Department of Food Science and Technology, The University of Agriculture Peshawar, Peshawar, Pakistan

**Keywords:** biochar, nitrogen, wheat, urea, farm yard manure and poultry manure

## Abstract

Crop quality and nutrient uptake are considerably influenced by fertilizers inputs and their application rate. Biochar (BC) improves nitrogen uptake and crop productivity. However, its interaction with synthetic and organic fertilizers in calcareous soil is not fully recognized. Therefore, we inspected the role of biochar (0, 10, 20, and 30 t ha^–1^) in improving N uptake and quality of wheat in a calcareous soil under integrated N management (90, 120, and 150 kg N ha^–1^) applied each from urea, farmyard manure (FYM) and poultry manure (PM) along with control) in 2 years field experiments. Application of 20 t BC along with 150 kg N ha^–1^ as poultry manure considerably improved wheat grain protein content (14.57%), grain (62.9%), straw (28.7%), and biological (38.4%) yield, grain, straw, and total N concentration by 14.6, 19.2, and 15.6% and their uptake by 84.6, 48.8, and 72.1%, respectively, over absolute control when averaged across the years. However, their impact was more pronounced in the 2nd year (2016–2017) after application compared to the 1st year (2015–2016). Therefore, for immediate crop benefits, it is recommended to use 20 t BC ha^–1^ once in 50 years for enhancing the nitrogen use efficiency of fertilizers and crop yield.

## Introduction

Agriculture is declared the main pillar of Pakistan’s economy, which greatly depends on the cereal crops (Rehman et al., 2015). Wheat (*Triticum aestivum* L.) being a staple food is the prominent cereal crop in Pakistan. It can be cultivated under different environments (Hussain et al., 2006) and provide the major nutritional needs (Khalil et al., 2002). However, wheat crop quality is majorly dependent upon the time, rate, and proportion of applied inputs especially nitrogenous fertilizers. Adequate N availability in soil and its uptake by the plants is very much critical for maintaining wheat production systems (Malhi et al., 2006; Wang et al., 2021). Brown and Petrie (2006) stated that optimum N availability has an encouraging impact on grain protein content (GPC) in wheat.

The use of chemical fertilizer is high compared to organic fertilizer because of the lower availability of crop nutrients in organic fertilizer (Han et al., 2016). Although, inorganic fertilizers enhance the initial mineralization of N, but they have been reported to decline soil quality by promoting soil acidification and compaction (Liu et al., 2010). However, their integrated application with organic materials for resolving such problems is documented in a study by Fageria and Baligar (2005). Integrated application of organic manures with synthetic fertilizer can help in maintaining soil fertility and productivity (Bandyopadhyay et al., 2010; Kumar et al., 2017). Poultry manure (PM), being an excellent organic manure, contains a high quantity of plant nutrients (Mussarat et al., 2021). Unlike chemical fertilizer, its addition into soil improves organic matter, promotes soil aggregation, water and nutrients holding capacity, aeration, and water infiltration (Deksissa et al., 2008). Similarly, FYM as an organic supplement improves soil physical and chemical fertility and, thus, encourages crop N uptake and GPC (Muqaddas et al., 2005; Silva et al., 2006; Khan et al., 2020). Though using PM and FYM as an alternative to chemical fertilizers is well documented, but it must be applied for reducing soil N losses. Additionally, the quantity to be applied depends upon soil condition, environmental conditions, and crop type.

Biochar (BC) improves soil N nutrition and its uptake by plants by preventing soil degradation via promoting soil aggregation, porosity, and water holding capacity under tropical conditions (Lehmann et al., 2003; Islami et al., 2011). Biochar application considerably enhanced N concentration in wheat leaves (24%), shoot/stem (20%), straw (24%), and grain (56%), while the protein content of grain (20%) (Ali et al., 2015). Typically BC has a strong adsorption capacity for nitrate and ammonium in water treatment and soil applications (Shenbagavalli and Mahimairaja, 2012; Fidel et al., 2018). Thus, it enhanced the storage of ammonium N in soil (Taghizadeh-Toosi et al., 2012). Hence, it also induced higher N uptake in both the above and subterranean plant parts (Backer et al., 2017; Cao et al., 2019). The application of BC to soil may be amazingly helpful for rehabilitating soil fertility, as well as to invigorate the plant growth, and, therefore, plays a significant role in building up a sustainable approach in agriculture (El-Naggar et al., 2019; Rawat et al., 2019). However, its performance varies under different soil and climatic conditions. Thus, this study was executed to explore the role of BC in improving N availability from differnt N sources in calcareous soil under semi-arid climatic conditions while using wheat as a test crop.

## Materials and Methods

### Experimental Site

Two-year field study was conducted during 2015–2017 at Agriculture Research Station Swabi (34° 7′, 48′′ N and 72° 28′, 11′′ E), Khyber Pakhtunkhwa, Pakistan. This study area is warm, semi-arid, and temperate climate with 639 mm annual rainfall (Khan et al., 2020). The soil of the experimental site was alkaline calcareous, nonsaline, silty loam in texture, and deficient in N (0.002%), P (4.6 mg kg^–1^), and K (66 mg kg^–1^) as reported by Khan et al. (2020).

### Experimental Inputs

Acacia pruning’s wood BC was prepared by the method already described by Arif et al. (2021). The FYM and PM were purchased from the local dairy and poultry farm, respectively. A similar field and layout of the experiment were used for the 2nd year experiment (in 2016–2017). The N, P, and K contents were 0.87, 0.34, and 0.6% in FYM and 1.53, 0.97, and 0.9% in PM, respectively. The biochar used in the experiment was composed of 0.08% N and 0.112% P.

### Experimental Procedure

This study was carried out to explore the role of biochar (0, 10, 20, and 30 t ha^–1^) in enhancing N availability from different N sources, including urea, FYM, and PM each applied at the rate of 90, 120, and 150 kg N ha^–1^ along with control. The above treatments were arranged in a two factorial randomized complete block design with split plot arrangement, each replicated three times. Biochar was applied to the main plot, while N management was applied into subplot. The experimental field was two times plowed prior to sowing approximately to a depth of 30 cm using a common cultivator, followed by planking in order to breakdown the clods and at the same time level the field. A total of 120 subplots each with a size of (3 m × 4 m) were set by using traditional implements. A spacing of 0.5, 1, and 2 m was maintained between subplots, main plots, and replications, respectively. Wheat seeds of variety Pirsabak 2013 were sown at a seed rate of 120 kg ha^–1^ in 30 cm apart rows. The proposed rates of N were applied from urea, FYM, and PM based on their actual N contents. The organic sources were applied 1 month before the sowing of the crop. Biochar was applied at the time of seedbed preparation at once to the wheat crop, while its carry-over effect was examined in the second year. Urea was applied in split doses. Full dose of FYM and PM was applied a month prior to sowing during the first year of the experiment. In the second year half of the FYM and PM, it was applied with the assumption that half of the FYM and PM decomposes during the first year of the experiment. Basal doses of phosphorus (P) and potassium (K) were applied at the rate of 90 and 60 kg ha^–1^ as SSP and SOP, respectively, at the time of sowing.

### Procedure for Recorded Observations

Plants were harvested from central two rows of each subplot at maturity and were separated into leaves, stem, spike, and grain. Grain (GNC) and straw nitrogen content (SNC) were measured during both the consecutive seasons by Kjeldahl method (Westerman, 1990) through the following formula:


$$\begin{array}{c} N\%=\\ \frac{\left\{\left(\mathrm{Nitrogen}\mathrm{blank}\right)\text{x}\left(\mathrm{Normality}\mathrm{of}\mathrm{acids}\text{x}\mathrm{Volume}\mathrm{made}\text{x}N\mathrm{mol}.\mathrm{weight}\right)\right\}}{\mathrm{Weight}\,\mathrm{of}\,\mathrm{dry}\,\mathrm{sample}}\times 100 \end{array}$$

Total nitrogen content (TNC) was considered by adding both grain and straw N content. Similarly, total nitrogen uptake (TNU) by wheat was calculated by adding both grain and straw N uptake. GPC was worked out from percent N present in the grain. Moreover, GPC (1), grain nitrogen uptake (GNU) (2), straw nitrogen uptake (SNU) (3), grain yield (GY) (4), straw yield (SY) (5), and biological yield (BY) (6) were calculated by using the following formulas.


(1) GPcontent(%)=GrainN(%)×6.25

(2) $$\begin{array}{c} \text{Grain}/\mathrm{straw}\,N\,\mathrm{uptake}\,\left(\mathrm{kg}\,{\mathrm{ha}}^{-1}\right)=\\ \frac{\mathrm{Grain}/\mathrm{straw}\,N\,\left(g\,\mathrm{kg}-1\right)\,x\,\mathrm{Grain}/\mathrm{straw}\,\mathrm{yield}\,\left(\mathrm{kg}\,\mathrm{ha}-1\right)}{1000} \end{array}$$

(3) BY⁢(kg⁢ha-1)=wt⁢of⁢straw+grains⁢of⁢middle⁢ 4⁢rows⁢ofeach⁢subplotR⁢to⁢R⁢distance×R⁢length×No⁢of⁢R⁢harvested×10,000

wt stands for weight, while R stands for row/s.


(4) GY⁢(kg⁢ha-1)=wt⁢of⁢grains⁢of⁢middle⁢ 4⁢rows⁢in⁢each⁢subplotR⁢to⁢R⁢distance×R⁢length×No⁢of⁢R⁢harvested×10,000⁢m2

wt stands for weight, while R stands for row/s.


(5) $$\mathrm{Straw}\,\mathrm{Yield}\,\left(\mathrm{kg}\,{\mathrm{ha}}^{-1}\right)=\text{BY}-\mathrm{GY}$$

BY stands for biological yield, while GY stands for grain yield.

### Statistical Analysis

The collected data were analyzed for ANOVA by using the appropriate procedure used for two factorial RCB designs using split-plot arrangement. All the analyses were done using SPSS 20th edition. For the significant *F* test, the mean of different treatments was compared at 5% *p*-value using the least significant difference (LSD) test (Steel and Torrie, 1980).

## Results

### Grain Yield

Grain yield (GY) had shown significant differences (*p* < 0.05) in response to the application of both the biochar and N management (Table 1). Further, BC and N management had also shown a significant interaction effect on GY. With respect to years, the GY of wheat was significantly higher during the years 2016–2017 than 2015–2016. Generally, the treatment which contains BC as a soil amendment overcomes the other treatment (control) in terms of GY. Specifically, maximum GY (13.26% over control) of wheat was obtained at 20 t BC ha^–1^, which was statistically at par to 30 t BC ha^–1^, while the lowest GY was produced by control.

**TABLE 1:** Effect of biochar and *n* levels applied from different sources on wheat grain, straw, and biological yield (kg ha^–1^).

Biochar (t ha^–1^)	GY	SY	BY
0	3,527 c	8,027 d	11,554 d
10	3,640 b	8,737 c	12,377 c
20	3,994 a	9,276 a	13,270 a
30	3,700 b	8,853 b	12,553 b
**LSD_(0.05)_**	**110.59**	**72.24**	**135.96**
**Nitrogen Management (kg ha^–1^)**			
Control	2,777 f	8,257 c	11,034 f
90 as Urea	3,420 e	8,801 ab	12,221 e
120 as Urea	3,742 cd	8,727 b	12,469 d
150 as Urea	4,065 a	8,739 b	12,804 ab
90 as FYM	3,727 cd	8,778 ab	12,505 d
120 as FYM	3,698 d	8,865 a	12,563 cd
150 as FYM	3,986 ab	8,742 b	12,728 abc
90 as PM	3,695 d	8,800 ab	12,495 d
120 as PM	3,887 bc	8,738 b	12,625 bcd
150 as PM	4,157 a	8,785 ab	12,942 a
**LSD_(0.05)_**	**174.86**	**114.21**	**214.98**
2015–2016	3,594 b	8,670 b	12,265 b
2016–2017	3,837 a	8,776 a	12,613 a
**Interactions**	**P_0.05_**	**P_0.05_**	**P_0.05_**
**BC × N**	Figure 1	Figure 2	Figure 3

*GY, grain yield, SY, straw yield; BY, biological yield; N, nitrogen; PM, poultry manure; FYM, farmyard manure. Values with different alphabets in each category are statistically different at 5% probability.*

**FIGURE 1:**
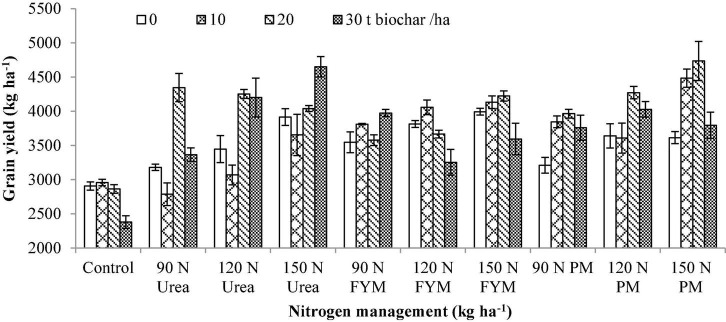
Interactive effect of biochar and *N* levels applied from different sources on grain yield of wheat. Bars on the graph denote SE of mean (*n* = 3). FYM, farmyard manure; PM, poultry manure.

**FIGURE 2:**
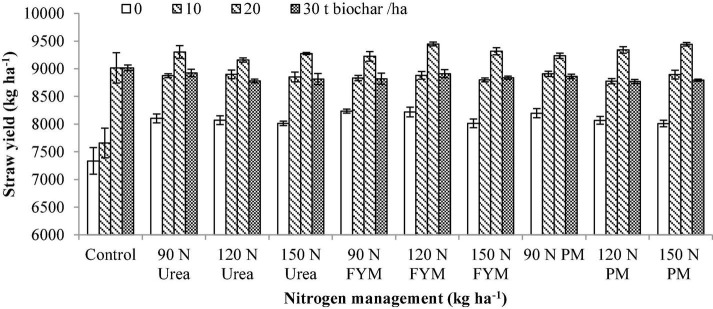
Interactive effect of biochar and *N* levels applied from different sources on straw yield of wheat. Bars on the graph denote the SE of mean (*n* = 3). FYM, farmyard manure; PM, poultry manure.

**FIGURE 3:**
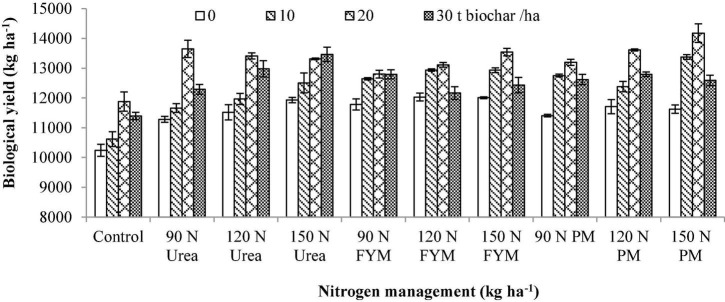
Integrated impact of biochar and *N* levels applied from different sources on biological yield of wheat. Bars on the graph denote SE of mean (*n* = 3). FYM, farmyard manure; PM, poultry manure.

As regard to the N management, the GY was significantly increased by (49.73%) over control by 150 kg N ha^–1^ as PM which was at par with 150 kg N ha^–1^ as urea (46.40% increase over control) while, significantly higher from the rest of the treatments. The lowest GY was produced by N control plots. Interaction between BC × N showed that plots amended with 20 t BC combined with 150 kg N as PM produced maximum GY (Figure 1).

### Straw Yield

The application of BC, N management, and their interaction significantly affected (for *p* < 0.05) wheat straw yield (SY) as given in Table 1. With regard to years, the SY of the wheat crop was significantly higher during the second year (2016–2017). Comparing the BC levels means, the maximum SY of (15.56% over control) was obtained at 20 t BC ha^–1^, which was followed by 30 and 10 t BC ha^–1^ showing 10.29 and 8.85% increase over control biochar, respectively. With respect to the influence of N management, the maximum SY (7.36% over control) was obtained at 120 kg N ha^–1^ as FYM. This was followed by 150, 150, 120, and 120 kg N ha^–1^ as FYM, urea, PM, and urea, having an increase of 5.87, 5.84, 5.83, and 5.70% over control, respectively. Moreover, the SY produced by 120 kg N as FYM was comparable to 90, 90, 150, and 90 kg N applied as urea, PM, PM, and FYM representing 6.59, 6.58, 6.40, and 6.31% increase over control N, respectively. Interaction between BC × N showed that plots amended with 20 t BC along with 120 kg N ha^–1^ as FYM produced maximum SY compared to the rest of the treatments combination, as shown in Figure 2.

### Biological Yield

The results of BY (Table 1) have disclosed the fact that BC and N addition had significantly improved BY of wheat. Further, BC and N management had also a significant interaction effect on GY. With regard to years, the BY was significantly higher during the years 2016–2017. Comparing the BC levels means, maximum BY (14.86% over control) was obtained at 20 t BC ha^–1^, followed by 30 and 10 t representing an increase of 8.65 and 7.13% over control BC plots, respectively.

With regard to N management from various sources, maximum BY was produced by 150 kg N ha^–1^ as PM with an increment of 17.30% over control, which was statistically at par to 150 kg N as urea showed 16.05% increase over control. The minimum BY was observed for N control plots. Interactively, 20 t BC combined with 150 kg N as PM produced maximum BY compared to the other combinations (Figure 3).

### Grain Nitrogen Content

**FIGURE 4:**
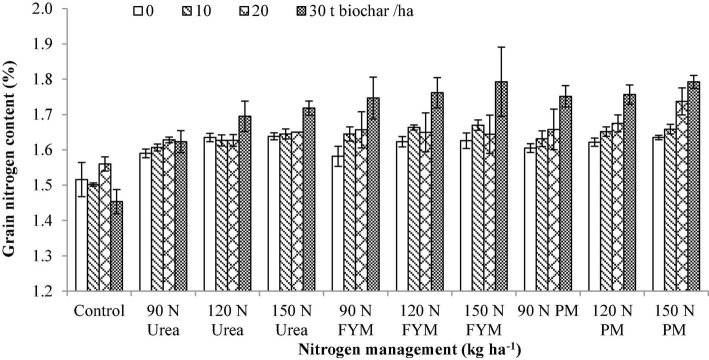
Interactive effect of biochar and N levels applied from different sources on grain nitrogen content of wheat. Bars on the graph denote SE of mean (*n* = 3). FYM, farmyard manure; PM, poultry manure.

Analysis of variance revealed that GNC significantly varied (*p* < 0.05) in response to the application of BC and nitrogen management (N). Interaction between BC and N was also significant for GNC. Moreover, significant variations were also noted for the years and those were higher during 2016–2017 than 2015–2016 (Table 2). Among the different BC levels, the highest GNC (6.16% increase over control) was observed at 30 t BC ha^–1^ followed by 20 t BC ha^–1^ (2.39% increase over control). The lower GNC was found in control BC plots. Among the different N levels applied from various sources, the highest GNC (12.97% increase over control) was observed at 150 kg N as PM. This was followed by 150 kg N as FYM, 120 kg N as PM FYM with an increase of 11.47, 11.01, and 10.89% over control, respectively. Interaction between BC × N indicated that plants treated with 30 t BC combined with 150 kg N as FYM and PM produced more GNC compared to the other treatments combinations (Figure 4).

**TABLE 2:** Nitrogen nutrition and grain protein content (%) of wheat as affected by biochar application under integrated *N* management.

Biochar (t ha^–1^)	GNC	SNC	TNC	GPC
0	1.61 c	0.43 c	2.033 c	10.045 c
10	1.63 bc	0.46 b	2.090 b	10.188 bc
20	1.65 b	0.46 b	2.110 b	10.303 b
30	1.71 a	0.50 a	2.213 a	10.683 a
**LSD_(0.05)_**	**0.02**	**0.01**	**0.03**	**0.27**
**Nitrogen Management (N kg ha^–1^)**				
Control	1.51 d	0.44 c	1.949 e	9.423 d
90 as Urea	1.61c	0.45 bc	2.062 d	10.075 c
120 as Urea	1.65 bc	0.45 bc	2.095 cd	10.286 bc
150 as Urea	1.66 b	0.45 bc	2.116 bc	10.393 b
90 as FYM	1.66 b	0.46 b	2.114 bc	10.359 b
120 as FYM	1.67 ab	0.47 a	2.148 ab	10.465 ab
150 as FYM	1.68 ab	0.48 a	2.161 a	10.520 ab
90 as PM	1.66 b	0.48 a	2.143 ab	10.385 b
120 as PM	1.68 ab	0.47 a	2.148 ab	10.477 ab
150 as PM	1.71 a	0.48 a	2.181 a	10.661 a
**LSD_(0.05)_**	**0.04**	**0.01**	**0.04**	**0.25**
2015–2016	1.6 b	0.4 b	2.043 b	10.1 a
2016–2017	1.7 a	0.5 a	2.181 a	10.5 b
**Interactions**	**P_0.05_**	**P_0.05_**	**P_0.05_**	**P_0.05_**
**BC × N**	Figure 4	Figure 5	Figure 6	Figure 7

*GNC, grain nitrogen content; SNC, straw nitrogen content; TNC, total nitrogen content; GPC, grain protein content; N, nitrogen; PM, poultry manure; FYM, farmyard manure. Values followed by different alphabets in each category are statistically different at 5% probability.*

**FIGURE 5:**
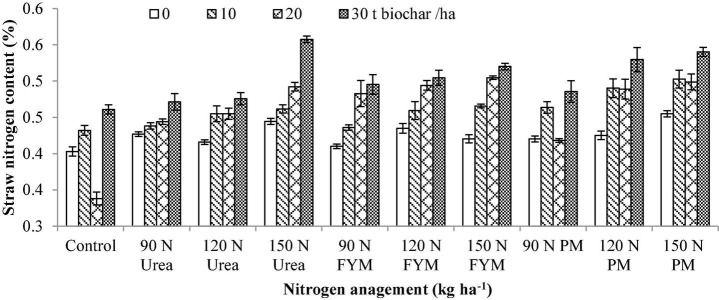
Interactive effect of biochar and N levels applied from different sources on straw nitrogen content of wheat. Bars on graph denote SE of mean (*n* = 3). FYM, farmyard manure; PM, poultry manure.

**FIGURE 6:**
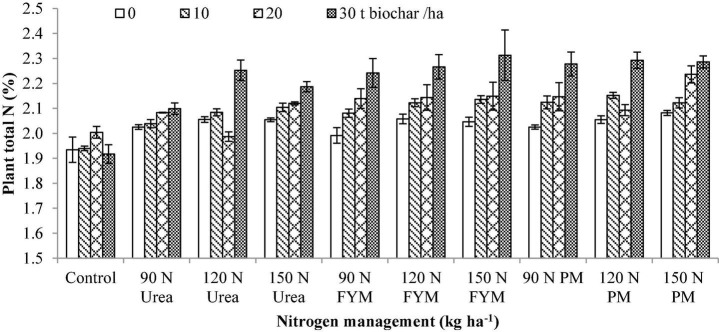
Interactive effect of biochar and *N* levels applied from different sources on total nitrogen of wheat. Bars on graph denote SE of mean (*n* = 3). FYM, farmyard manure; PM, poultry manure.

**FIGURE 7:**
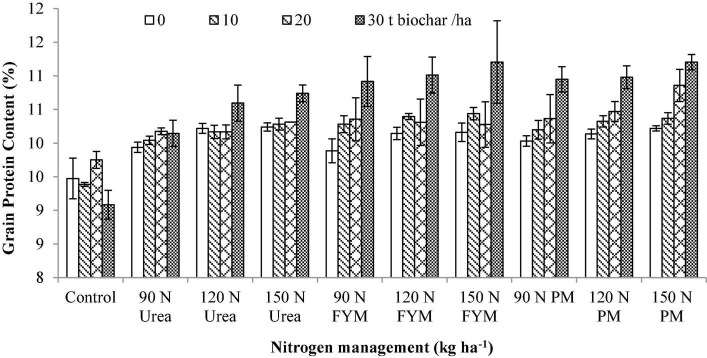
Interactive effect biochar and *N* levels applied from different sources on the grain protein content of wheat. Bars on graph denote SE of mean (*n* = 3). FYM, farmyard manure; PM, poultry manure.

### Straw Nitrogen Content

The results of this study indicate that straw nitrogen content (SNC) significantly varied (*p* < 0.05) in response to the application of BC and N management. Interaction between BC and N was also significant for SNC. Moreover, significant variations were also noted for the years and those were higher during 2016–2017 than 2015–2016 (Table 2). Among different BC levels, the highest SNC was noted under 30 t BC ha^–1^ having an increase of 17.23% over control. This was followed by 20 and 10 t BC ha^–1^ with an increase of 7.36 and 7.08%, respectively over control; however, their impact was at par to one another but was more than control. Similarly, the highest SNC was noted at 90 kg N ha^–1^ as PM with an increase of 9.51% over control. This was followed by 150, 150, 120, and 120 kg N ha^–1^ applied as FYM, PM, FYM, and PM having an increase of 8.85, 8.10, 7.55, and 7.7%, respectively, over control. However, their differences were at par to each other and significantly higher than the control. Interaction between BC × N indicated that plants treated with 30 t BC along with 120 kg N applied from PM ha^–1^ produced more SNC than the rest of the treatments (Figure 5).

### Total Nitrogen Content

Results from this study showed that total nitrogen content (TNC) significantly varied (*p* < 0.05) in response to the application of BC and N management. Interaction between BC and N was also significant for TNC. Moreover, significant variations were also noted for the years and those were higher during 2016–2017 than 2015–2016 (Table 2). Comparison different BC levels, the highest TNC was recorded for 30 t BC ha^–1^ where 8.87% increase over control was observed followed by 20 and 10 t BC having with an increase of 3.80 and 2.83% over control. Similarly, the highest TNC was observed at 150 kg N as PM exhibiting 11.93% increase over control followed by 150 kg N as FYM, 120, 90 kg N as PM, and 120 kg N as FYM with an increase of 10.88, 10.22, 9.97, and 10.19%, respectively over control, while significantly lower TNC was observed in control plots. Generally, BC × N indicated that the highest TNC was observed in plots treated with 30 t BC along with 150 kg N ha^–1^ was applied from FYM (Figure 6).

### Grain Protein Content

Our results showed that GPC significantly varied (*p* < 0.05) in response to the application of BC and N management. Interaction between BC and N was also significant for GPC. Moreover, significant variations were also noted for the years and those were higher during 2016–2017 than 2015–2016 (Table 2). Significantly higher GPC was noted at 30 t BC ha^–1^, where 7% over control BC was observed. This was followed by 20 t BC showing 6.00% over control. The result obtained at 20 t BC was statistically at par with 10 t BC; however, still an increase of 4.00% was observed over control. For N management, the highest GPC was observed at 150 kg N ha^–1^ as PM, showing an increase of 13.18% over control. The GPC obtained at 150 kg N as FYM did not show any significant differences when compared to 120 kg N applied from PM and FYM; however, still an increase of 11.22 and 11.09% was observed over control. Moreover, 150 kg N from urea did not show any significant differences when compared to (90 kg N from PM and FYM), where an increase of 10.24 and 9.97% were observed over control. Interaction between BC × N indicated that plots amended with 30 t BC produced the highest GPC when combined with 150 kg N ha^–1^ as PM and urea (Figure 7).

### Grain Nitrogen Uptake

**FIGURE 8:**
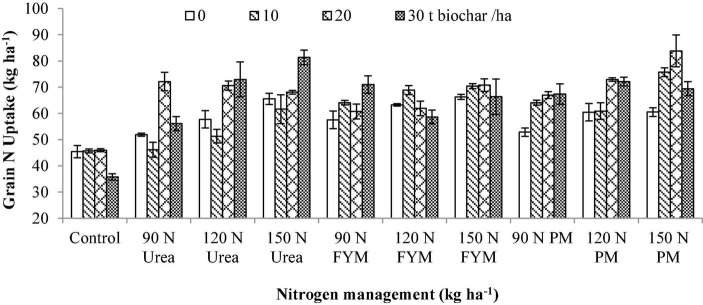
Interactive effect biochar and *N* levels applied from different sources on grain nitrogen uptake of wheat. Bars on graph denote SE of mean (*n* = 3). FYM, farmyard manure; PM, poultry manure.

The variation in GNU of wheat as influenced by BC and N management is given in Table 3. The results showed that GNU significantly varied (*p* < 0.05) in response to the application of BC and N management. Interaction between BC and N was also significant for GNU. Moreover, significant variations were also noted for the years and those were higher during 2016–2017 than 2015–2016 (Table 3). While comparing mean GNU of various BC levels, the highest GNU was observed at 20 t BC ha^–1^ producing a 15.92% increase over control followed by 30 and 10 t BC where 12.0 and 4.71% increase over control were observed, respectively. Similarly, the higher GNU was observed at 150 kg N ha^–1^ as PM, showing an increase of 67.51% over control. Furthermore, the 150 kg N as urea and PM did not show any significant differences when compared to each other. Interaction between BC × N indicated that plots amended with 20 t BC resulted in maximum GNU when combined with 150 kg N applied from PM (Figure 8).

**TABLE 3:** Grain, straw, and total nitrogen uptake (kg ha^–1^) in wheat as affected by biochar and nitrogen application from different sources.

Biochar (t ha^–1^)	GNU	SNU	TNU
0	58.14 d	34.18 d	92.32 c
10	60.88 c	40.28 c	101.15 b
20	67.39 a	42.88 b	110.28 a
30	65.11 b	44.63 a	109.75 a
**LSD_(0.05)_**	**2.54**	**0.88**	**2.45**
**Nitrogen Management (kg ha^–1^)**			
Control	43.20 f	36.55 d	79.75 f
90 as Urea	56.58 e	39.61 c	96.19 e
120 as Urea	63.15 cd	39.15 c	102.31 d
150 as Urea	69.14 ab	39.76 c	108.90 bc
90 as FYM	63.34 cd	40.15 bc	103.49 d
120 as FYM	63.17 cd	42.07 a	105.24 cd
150 as FYM	68.46 b	41.94 a	110.40 ab
90 as PM	62.82 d	42.54 a	105.37 cd
120 as PM	66.60 bc	41.25 ab	107.85bc
150 as PM	72.37 a	41.90 a	114.26 a
**LSD_(0.05)_**	**3.61**	**1.39**	**3.87**
2015–2016	61.3 b	36.37 b	97.69 b
2016–2017	64.4 a	44.61 a	109.06 a
**Interactions**	**P_0.05_**	**P_0.05_**	**P_0.05_**
**BC × N**	Figure 5	Figure 6	Figure 7

*GNU, grain nitrogen uptake; SNU, straw nitrogen uptake; TNU, total nitrogen uptake; N, nitrogen; PM, poultry manure; FYM, farmyard manure. Values followed by different alphabets in each category are statistically different at 5% probability.*

### Straw Nitrogen Uptake

**FIGURE 9:**
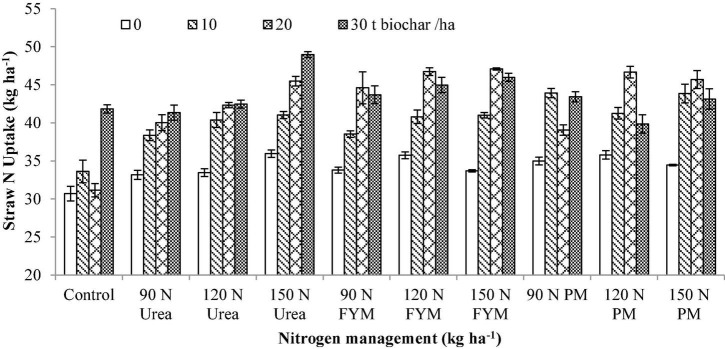
Interactive effect biochar and *N* levels applied from different sources on straw nitrogen uptake of wheat. Bars on graph denote SE of mean (*n* = 3). FYM, farmyard manure; PM, poultry manure.

Wide variations were recorded for SNU due to BC and N application (Table 3). The results showed that SNU significantly varied (*p* < 0.05) in response to the application of BC and N management. Interaction between BC and N was also significant for SNU. Moreover, significant variations were also noted for the years and those were higher during 2016–2017 than 2015–2016. Significantly higher SNU was observed at 30 t BC, having an increase of 30.58% over control. This was followed by 20 and 10 t BC ha^–1^ having an increase of 25.47 and 17.84%, respectively, over control. For N management, the higher SNU was observed where 90 kg N as PM with an increase of 16.39% over control. The SNU observed at 90 kg N as PM was at par to 120, 150 kg N as FYM and with 120, 150 kg N as PM. However, still an increase of 15.09, 14.75, 12.86, and 14.63% was observed, respectively, over control. The SNU observed at 150 kg N ha^–1^ as urea was also at par to 90 and 120 kg N from urea. Generally, BC × N indicated that the highest SNU was observed in those plots where 30 t BC and 120 kg N ha^–1^ were applied as urea; however, statistically similar results were also observed at 20 t BC along with 150 kg N applied from PM (Figure 9).

### Total Nitrogen Uptake

**FIGURE 10:**
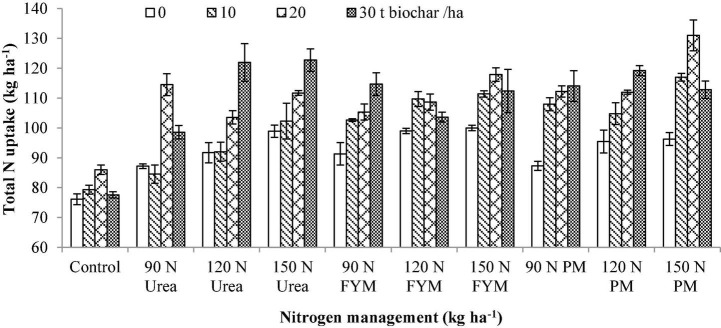
Interactive effect of biochar and *N* levels applied from different sources on total nitrogen uptake by wheat. Bars on graph denote SE of mean (*n* = 3). FYM, farmyard manure; PM, poultry manure.

Application of both the BC and N significantly influenced total nitrogen uptake (TNU) by wheat. The interactive effect between BC and N was also significant for TNU. Moreover, significant variations were also noted for the years and those were higher during 2016–2017 than 2015–2016 (Table 3). The highest TNU was observed under 20 t BC(with an increase of 19.45% over control), which was at par to 30 t BC (with an increase of 18.88% over control) followed by 10 t BC (with an increase of 9.57%) over control. In the case of N management, the higher TNU was observed at 150 kg N as PM, representing 43.28% increase over control. However, it was statistically similar to 150 kg N as FYM, representing 38.43% improvement over control. The lowest TNU was observed at control N plots. Interactively, 20 t BC was applied along with 150 kg N ha^–1^ as PM showed the highest TNU (Figure 10).

## Discussion

### Wheat Yield as Influenced by Biochar Under Integrated N Management

Application of both the biochar and nitrogen, irrespective of their sources, significantly increased wheat yield; however, their impact was more pronounced when 20-ton biochar was applied with 150 kg N ha^–1^ as PM. The increase in GY under biochar amended plots might be due to the nutritive nature of BC that enriches the soil with essential nutrients for the long run, reduce nutrients leaching, and ultimately boost up soil fertility. This is in confirmation with a study by Mierzwa-Hersztek et al. (2019) who revealed that BC enhanced GY by improving soil physical, chemical, and biological properties, and ultimately improving crop growth and yield. Similarly, Uzoma et al. (2011) reported that application of 30 and 20 t BC ha^–1^ considerably enhanced the GY of maize when compared to control. Our results are consistent with those found by other researchers. Park et al. (2005) who observed that BC improved the mineralization of organic matter (OM) in the soil, which further had a positive effect on crop growth and ultimately on yield. Minimum SY in no BC treated plot might be due to the lower nutrient concentration in these plots. Enhanced SY of wheat in BC incorporated plots was noted by Sarma et al. (2017) and maximum SY of maize by Faloye et al. (2017). Peng et al. (2011) and Faloye et al. (2017) also noted similar improvements in stover yield of maize in BC incorporated plots. Alburquerque et al. (2014) also observed the highest dry biomass of sunflower at a high rate of BC made from olive tree pruning and is mainly due to its nutritive nature and poor fertility of the soil used during the experiment. An increase in BY due to BC incorporation might be due to its indirect effect by reducing nutrients leaching and enhancing the use efficiency of fertilizer (Lehmann et al., 2003; Liu et al., 2017). Similarly, Zhang et al. (2012) observed in their study that the application of BC improved cereal yield and its components. They attributed it to enhancement in texture and structure of the soil due to C and N organic sources. BC attracts ammonium and nitrate ions; hence, increasing N concentration in the soil which further improved vegetative growth and ultimately total biomass is improved (Steiner et al., 2007; Genesio et al., 2012). Van et al. (2010) observed a positive response of crop yield due to the sole use of BC, which are possibly due to its nature (rich in carbon and nutrient-poor material).

We observed maximum wheat grain yield (GY) at 150 kg N ha^–1^ applied as PM and urea. N application might have enhanced photosynthesis which resulted in greater yield in response to a greater amount of dry matter and assimilates production and its translocation to the seed. In conformity to our findings, Elli et al. (2015) noted that increasing levels of N as urea increased the amount of available N for plants which further increase the LA, LAI, and photosynthesis that eventually maximized the GY of wheat. The improvement in GY through PM incorporation might be due to the maximum available nutrients in the soil and their effective consumption by the crop plants. Boateng et al. (2006) and Ayoola and Makinde (2009) obtained similar improvement in GY through the application of PM. This increase in total biomass may possibly be because N supply enhances LA, LAI, leaf chlorophyll content, and overall vegetative growth. The above statement is further supported by Salem et al. (2011). Ali et al. (2011) also reported maximum shoot dry weight in N applied plots may possibly be due to improved number of tillers, leaves, and LA which further leads to enhanced photosynthetic activity. Jan et al. (2011) observed substantial improvement in wheat grain as well as straw yields through incorporation FYM to inorganic fertilizers when compared with FYM control plots. We have found that application of 150 kg N solely from PM, urea, and FYM significantly increased BY of wheat. Because the higher application of N had improved LA, LAI, plant height, grain yield, tillers m^–2^, and total dry matter which collectively give rise to BY. The greater BY in organic manures amended plots may possibly be due to more moisture and nutrients availability hence kept better growth throughout the growing period (Abbas et al., 2012), thus resulted in greater biomass production (Khan et al., 2018). The other possible reasons might be the addition of inorganic fertilizer as well as mineralization of organic manures that did not expose the plants to nutrient stress at any point during the entire growing season and thus lead to a higher total biomass production (Mazhar et al., 2018). Compared to our results, Jan et al. (2018) observed more BY in plots incorporated with PM. This was due to the greater nutrient concentrations in PM when compared to other organic sources.

### Wheat N Concentration and Uptake as Influenced by Biochar Under Integrated N Management

Application of BC significantly increased wheat GNC and SNC of wheat compared to control. The reason could be the higher nutrients availability in BC incorporated plots which further improves plant growth and quality attributes due to the high N uptake. Ali et al. (2015) also noted 56% improvement in GNC in response to 25 t BC ha^–1^. Similarly, Major et al. (2010) also stated that N content in maize grains was improved due to BC incorporation. While Ali et al. (2015) noted that SNC of wheat was considerably enhanced (24%) due to BC incorporation at 25 t ha^–1^. Major et al. (2010) also observed higher SNC in BC incorporated plots at 20 t ha^–1^. Compare to our results DeLuca et al. (2009) noted that incorporation of BC improved plant N content which further increased the use efficiency of N. In this study wheat GPC was significantly enhanced due to BC application. It might be due to extend stay of NH4^+^ ion in soil, probably due to BC because BC holds ammonium ion and makes it inaccessible for microbes to transform it into NO_3_ and considerably minimizes losses of N due to volatilization and leaching in soil. These results were sustained by Major et al. (2010) who noted that application of 20 t BC ha^–1^ considerably enhanced N uptake in maize, due to its direct adsorption effect and/or indirectly through microbial immobilization effect. Lehmann et al. (2003) observed an enhanced percentage of nitrogen contents in grains wheat due to the incorporation of BC amended urea, hence considerably improved GPC in wheat (Ercoli et al., 2013; Wan et al., 2014). Ali et al. (2015) also observed a 20% improved GPC in wheat in response to 25 t biochar ha^–1^. This increase in GNU and SNU uptake of wheat under BC amended plots confirmed the valuable effect of BC to increase fertilizer use efficiency especially in soils where N loss is a main environmental and agronomic concern. The improvement in GNU is accredited due to the release of nutrients (mostly N) by BC (Zheng et al., 2013; Ahmad et al., 2022), also, BC helps in reducing nutrients run-off, leaching, and holding moisture (Chan et al., 2008; Steiner et al., 2008), improve root growth (Torii, 2012) resulted in improved nutrient availability for plants, and finally increase nutrient uptake was observed. Abukari et al. (2018) also stated that this is possibly due to the binding nature of BC, which reduces N losses. Thus, the enhanced nutrient retaining capability of BC further improves dry matter production indicating greater nutrient uptake. Alike result were also obtained by Backer et al. (2017), Cao et al. (2019) who observed higher nutrient N uptake in root and shoot of wheat in BC amended plots.

Improvement in wheat N nutrition and grain quality with Biochar and N supplementation could be possibly associated with optimum availability of moisture and N in soil that can enhance chlorophyll contents and rate of photosynthesis as a result produce grains with higher protein content. Organic sources of N improve organic matter content in the soil (Maltas et al., 2018), increase N availability (Dikinya and Mufwanzala, 2010), and its uptake (Shaji et al., 2021), increase chlorophyll contents (Hussain et al., 1988), improve LA (Ahmed et al., 2017), and rate of photosynthesis (Shah et al., 2010), hence maximum assimilates are partition into the grain (Meng et al., 2005). Our results are in accordance with the findings of Shah and Ahmad (2006) who observed greater N content in wheat straw through the application of FYM. Conacher and Conacher (1998) found greater N uptake, (Kashif et al., 2018; Muhammad et al., 2019) and reduced NO_3_-N loses in PM amended soil. Ghoneim (2007), Zhang et al. (2016) found higher N uptake due to the application of PM and FYM. The improvement in seed quality (especially GPC) through the use of the organic source of fertilizer is associated with the increased availability of essential nutrients to the crop plants. Zenawi and Mizan (2019) confirmed that N contents of leaves are promptly transformed to protein and during seed development leaf N is converted to seed for protein synthesis. Greater LAI with the same level of PM might be one of the key causes for these greater protein contents (Khan et al., 2018). Abbasi and Khaliq (2016) noted positive response of PM compared to other sources due to a greater percentage of N in the available form in PM. Singh et al. (2013) stated that incorporation of FYM considerably improved N uptake and grain and straw yields of wheat and protein content in its grain. The increase of N content in grain may be due to the effective use of all the available N (Yaduvanshi and Swarup, 2005) supplied by various sources (Jagadeeswari and Kumaraswamy, 2000). The higher N uptake by wheat was due to the higher availability of N in the adequate amount supplied steadily by organic manures throughout the growing season. Chaudhry et al. (2013) stated that quantity, quality, and types of organic manures positively affect the supply of nutrients to crops, while Diacono and Montemurro (2011) observed that different sources of organic amendments have different decomposition rates; as a result, the availability of nutrients from these manures (through the process of mineralization) and uptake of nutrients by the crop vary. The incorporation of organic N sources improves soil water retaining capability, which further contributed to efficient uptake of nutrients (Adnan et al., 2016; Shaji et al., 2021). This statement is further supported by Shah et al. (2012) who also observed greater N uptake by wheat grains. Similarly, Woldesenbet and Haileyesus (2016) found greater NP uptake by barely straw through the application of FYM at (5 t ha^–1^). Further, Bodruzzaman et al. (2010), Islam et al. (2016) also confirmed increase nutrient uptake by wheat straw was positively influenced by organic amendments. Reganold (1995) found greater N uptake (Kashif et al., 2018) and reduced NO_3_-N losses in PM amended soil. Our result is further supported by Ghoneim (2007), Zhang et al. (2016) who found higher N uptake due to applications of PM and FYM.

## Conclusion

The application of 20 t BC along with 150 kg N ha^–1^ as PM significantly improved wheat grain (62.9%), straw (28.7%), and biological (38.4%) yield, grain, straw, and total N concentration by 14.6, 19.2, and 15.6%, while their uptake by 85, 49, and 72%, respectively, over absolute control when averaged across the years. The impact of applied treatments especially biochar and organic N sources were more pronounced in the 2nd year after application than the 1st year. Hence, the application of 20 t biochar ha^–1^ once in 50 years along with 150 kg N ha^–1^ as PM is a promising option for improving nutrient uptake and crop quality in semi-arid climatic conditions under calcareous soils.

## Data Availability Statement

The raw data supporting the conclusions of this article will be made available by the authors, without undue reservation.

## Author Contributions

AB: conceptualization and supervision. AA-H, MK, MA, MS, AI, TN, and Amanullah: methodology and formal analysis. AA-H and SF: writing—original draft preparation. SS, KL, MH, and SF: writing—review and editing. AA-H: funding acquisition. All authors contributed to the article and approved the submitted version.

## Conflict of Interest

The authors declare that the research was conducted in the absence of any commercial or financial relationships that could be construed as a potential conflict of interest.

## Publisher’s Note

All claims expressed in this article are solely those of the authors and do not necessarily represent those of their affiliated organizations, or those of the publisher, the editors and the reviewers. Any product that may be evaluated in this article, or claim that may be made by its manufacturer, is not guaranteed or endorsed by the publisher.

## References

[B1] Abbas G. Khattak J. Z. K. Mir A. Ishaque M. Hussain M. Wahedi H. M. (2012). Effect of organic manures with recommended dose of NPK on the performance of wheat (*Triticum aestivum* L.). *J. Anim. Plant Sci.* 22 683–687.

[B2] Abbasi M. K. Khaliq A. (2016). Nitrogen mineralization of a loam soil supplemented with organic inorganic amendments under laboratory incubation. *Front. Plant Sci.* 7:1038. 10.3389/fpls.2016.01038 27493649 PMC4954816

[B3] Abukari A. Abunyewa A. A. Issifu H. (2018). Effect of rice husk biochar on nitrogen uptake and grain yield of maize in the guinea savanna zone of ghana. *Int. J. Dev.* 5 1–6. 10.9734/jeai/2018/41305

[B4] Adnan M. Shah Z. Arif M. Khan M. J. Mian I. A. Sharif M. (2016). Impact of rhizobial inoculum and inorganic fertilizers on nutrients (NPK) availability and uptake in wheat crop. *Can. J. Soil Sci.* 96 169–176. 10.1139/cjss-2016-0012

[B5] Ahmad W. Khan A. Zeeshan M. Ahmad I. Adnan M. Fahad S. (2022). Relative efficiency of biochar particles of different sizes for immobilising heavy metals and improving soil properties. *Crop Pasture Sci.* 10.1071/CP20453

[B6] Ahmed B. E. A. M. Ishag A. A. Hassan M. K. Ahmed M. O. (2017). Response of two wheat cultivars (*Triticum aestivum* L.) to amended nitrogen fertilizer on yield and grain quality in Halfa Elgadidah area. *MOJ Biol. Med.* 1:00029.

[B7] Alburquerque J. A. Calero J. M. Barrón V. Torrent J. Del Campillo M. C. Gallardo A. (2014). Effects of biochars produced from different feedstocks on soil properties and sunflower growth. *J. Plant Nutr. Soil Sci.* 177 16–25. 10.1002/jpln.201200652

[B8] Ali K. Arif M. Jan M. T. Khan M. J. Jones D. L. (2015). Integrated use of biochar: a tool for improving soil and wheat quality of degraded soil under wheat-maize cropping pattern. *Pak. J. Bot.* 47 233–240.

[B9] Ali K. Munsif F. Zubair M. Hussain Z. Shahid M. Din I. U. (2011). Management of organic and inorganic nitrogen for different maize varieties. *Sarhad J. Agric*. 27 525–529.

[B10] Arif M. Ali S. Ilyas M. Riaz M. Akhtar K. Ali K. (2021). Enhancing phosphorus availability, soil organic carbon, maize productivity and farm profitability through biochar and organic–inorganic fertilizers in an irrigated maize agro-ecosystem under semi-arid climate. *Soil Use Manage.* 37 104–119. 10.1111/sum.12661

[B11] Ayoola O. T. Makinde E. (2009). Maize growth, yield and soil nutrient changes with N-enriched organic fertilizers. *Afr. J. Food Agric. Nutr. Dev.* 9 580–592. 10.4314/ajfand.v9i1.19214

[B12] Backer R. G. Saeed W. Seguin P. Smith D. L. (2017). Root traits and nitrogen fertilizer recovery efficiency of corn grown in biochar amended soil under greenhouse conditions. *Plant Soil* 415 465–477. 10.1007/s11104-017-3180-6

[B13] Bandyopadhyay K. K. Misra A. K. Ghosh P. K. Hati K. M. (2010). Effect of integrated use of farmyard manure and chemical fertilizers on soil physical properties and productivity of soybean. *Soil Tillage Res.* 110 115–125. 10.1016/j.still.2010.07.007

[B14] Boateng S. Zickermann A. J. Kornaharens M. (2006). Effect of poultry manure on growth and yield of maize. *West Afr. J. Appl. Ecol.* 9 1–11. 10.4314/wajae.v9i1.45682

[B15] Bodruzzaman M. Meisner C. A. Sadat M. A. Hossain M. I. (2010). “Long-term effects of applied organic manures and inorganic fertilizers on yield and soil fertility in a wheat-rice cropping pattern,” in *Proceedings of the 19th World Congress of Soil Science*, Brisbane, QLD, 10–15.

[B16] Brown B. D. Petrie S. (2006). Irrigated hard winter wheat response to fall, spring, and late season applied nitrogen. *Field Crops Res.* 96 260–268. 10.1016/j.fcr.2005.07.011

[B17] Cao H. Ning L. Xun M. Feng F. Li P. Yue S. (2019). Biochar can increase nitrogen use efficiency of *Malus hupehensis* by modulating nitrate reduction of soil and root. *Appl. Soil Ecol.* 135 25–32. 10.1016/j.apsoil.2018.11.002

[B18] Chan K. Y. Van Zwieten L. Meszaros I. Downie A. Joseph S. (2008). Using poultry litter biochars as soil amendments. *Soil Res.* 46 437–444. 10.1071/SR08036

[B19] Chaudhry A. N. Naeem M. A. Jilani G. Razzaq A. Zhang D. M. Azeem M. (2013). Influence of composting and poultry litter storage methods on mineralization and nutrient dynamics. *J. Anim. Plant Sci.* 23 500–506.

[B20] Conacher J. Conacher A. (1998). Organic farming and the environment, with particular reference to Australia: a review. *Biol. Agric. Hortic.* 16 145–171. 10.1080/01448765.1998.9755229

[B21] DeLuca T. H. MacKenzie M. D. Gundale M. J. (2009). “Biochar effects on soil nutrient transformations,” in *Biochar for Environmental Management: Science and Technology*, eds Lehmann J. Joseph S. (London: Earthscan).

[B22] Deksissa T. Short I. Allen J. (2008). “Effect of soil amendment with compost on growth and water use efficiency of Amaranth,” in *Proceeding of the COWR/NTwR Annual Conference International Water Resources Challenges for the 21st Country and Water Resources Education July 22-24, 2008*, Durban, NC, 22–24.

[B23] Diacono M. Montemurro F. (2011). Long-term effects of organic amendments on soil fertility. *Int. Sustain. Agric.* 2 761–786. 10.1007/978-94-007-0394-0_34

[B24] Dikinya O. Mufwanzala N. (2010). Chicken manure enhanced soil fertility and productivity: effects of application rates. *J. Soil Sci. Environ. Manage.* 1 46–54. 10.5897/JSSEM.9000019

[B25] Elli E. F. Caron B. O. Medeiros S. L. P. Eloy E. Monteiro G. C. Schmidt D. (2015). Effects of growth reducer and nitrogen fertilization on morphological variables, SPAD index, interception of radiation and productivity of wheat. *Rev. Ceres* 62 577–582. 10.1590/0034-737X201562060010

[B26] El-Naggar A. El-Naggar A. H. Shaheen S. M. Sarkar B. Chang S. X. Tsang D. C. (2019). Biochar composition dependent impacts on soil nutrient release, carbon mineralization, and potential environmental risk: a review. *J. Environ. Manage.* 241 458–467. 10.1016/j.jenvman.2019.02.044 31027831

[B27] Ercoli L. Masoni A. Pampana S. Mariotti M. Arduini I. (2013). As durum wheat productivity is affected by nitrogen fertilisation management in Central Italy. *Eur. J. Agron.* 44 38–45. 10.1016/j.eja.2012.08.005

[B28] Fageria N. K. Baligar V. C. (2005). Enhancing nitrogen use efficiency in crop plants. *Adv. Agron.* 88 97–185. 10.1016/S0065-2113(05)88004-6

[B29] Faloye O. T. Michael A. Ajayi A. E. Ewulo B. S. (2017). Synergistic effects of biochar and inorganic fertiliser on maize (*Zea mays*) yield in an alfisol under drip irrigation. *Soil Tillage Res.* 174 214–220. 10.1016/j.still.2017.07.013

[B30] Fidel R. B. Laird D. A. Spokas K. A. (2018). Sorption of ammonium and nitrate to biochars is electrostatic and pH-dependent. *Sci. Rep.* 8:17627. 10.1038/s41598-018-35534-w 30514956 PMC6279760

[B31] Genesio L. Miglietta F. Lugato E. Baronti S. Pieri M. Vaccari F. P. (2012). Surface albedo following biochar application in durum wheat. *Environ. Res. Lett.* 7:014025. 10.1088/1748-9326/7/1/014025

[B32] Ghoneim A. (2007). Effect of nitrogen supplied from poultry manure and sewage sludge on growth, yield and nitrogen uptake of rice. *Bull. Exp. Farm Fac. Agric. Ehime Univ.* 29 11–16.

[B33] Han S. H. An J. Y. Hwang J. Kim S. B. Park B. B. (2016). The effects of organic manure and chemical fertilizer on the growth and nutrient concentrations of yellow poplar (*Liriodendron tulipifera* Lin.) in a nursery system. *For. Sci. Technol.* 12 137–143. 10.1080/21580103.2015.1135827

[B34] Hussain I. Khan M. A. Khan E. A. (2006). Bread wheat varieties as influenced by different nitrogen levels. *J. Zhejiang Univ. Sci.* 7 70–78. 10.1631/jzus.2006.B0070 16365929 PMC1361763

[B35] Hussain T. Jilani G. Iqbal M. Z. (1988). Integrated use of organic and inorganic N fertilizers in rice-wheat cropping system. *Pak. J. Soil Sci.* 3 19–23.

[B36] Islam M. Anwar S. Khan B. Shah W. A. Ali M. Uddin S. (2016). Effect of nitrogen fertilization and decapitation stress on wheat (*Triticum aestivum* L.) productivity. *Pure Appl. Biol.* 5 317–325. 10.19045/bspab.2016.50041

[B37] Islami T. Guritno B. Basuki N. Suryanto A. (2011). Biochar for sustaining productivity of cassava based cropping systems in the degraded lands of East Java, Indonesia. *J. Trop. Agric.* 49 40–46.

[B38] Jagadeeswari P. V. Kumaraswamy K. (2000). Long-term effects of manure-fertilizer schedules on the yield of and nutrient uptake by rice crop in a permanent manurial experiment. *J. Indian Soc. Soil Sci.* 48 833–836.

[B39] Jan A. Amanullah Noor M. (2011). Wheat response to farm yard manure and nitrogen fertilization under moisture stress conditions. *J. Plant Nutr.* 34 732–742. 10.1080/01904167.2011.540688

[B40] Jan M. F. Ahmadzai M. D. Liaqat W. Ahmad H. Rehan W. (2018). Effect of poultry manure and phosphorous on phenology, yield and yield components of wheat. *Int. J. Curr. Microbiol. Appl. Sci.* 7 3751–3760. 10.20546/ijcmas.2018.704.422

[B41] Kashif M. Javed M. Ullah S. Ali A. Khan G. R. (2018). Effect of planting methods and nitrogen sources on yield, yield components and N-uptake of spring maize. *Adv. Crop Sci. Technol.* 6:373. 10.4172/2329-8863.1000373

[B42] Khalil I. H. Carver B. F. Krenzer E. G. MacKown C. T. Horn G. W. Rayas-Duarte P. (2002). Genetic trends in winter wheat grain quality with dual-purpose and grain-only management systems. *Crop Sci.* 42 1112–1116. 10.2135/cropsci2002.1112 34798789

[B43] Khan M. A. Basir A. Saeed B. (2020). Biochar improves phenological and physiological attributes of wheat in soil amended with organic and inorganic nitrogen sources. *Sarhad J. Agric.* 36 1214–1226. 10.17582/journal.sja/2020/36.4.1214.1226

[B45] Khan T. U. Jan M. T. Khan A. Ahmad G. Ishaq M. Afridi K. (2018). Integrated management of fertilizer nitrogen and poultry manure enhances wheat production. *Pak. J. Agric. Res.* 31 207–215. 10.17582/journal.pjar/2018/31.3.207.215

[B46] Kumar U. Shahid M. Tripathi R. Mohanty S. Kumar A. Bhattacharyya P. (2017). Variation of functional diversity of soil microbial community in sub-humid tropical rice-rice cropping system under long-term organic and inorganic fertilization. *Ecol. Indic.* 73 536–543. 10.1016/j.ecolind.2016.10.014

[B47] Lehmann J. da Silva J. P. Steiner C. Nehls T. Zech W. Glaser B. (2003). Nutrient availability and leaching in an archaeological Anthrosol and a Ferralsol of the Central Amazon basin: fertilizer, manure and charcoal amendments. *Plant Soil* 249 343–357. 10.1023/A:1022833116184

[B48] Liu E. Yan C. Mei X. He W. Bing S. H. Ding L. (2010). Long-term effect of chemical fertilizer, straw, and manure on soil chemical and biological properties in northwest China. *Geoderma* 158 173–180. 10.1016/j.geoderma.2010.04.029

[B49] Liu Z. He T. Cao T. Yang T. Meng J. Chen W. (2017). Effects of biochar application on nitrogen leaching, ammonia volatilization and nitrogen use efficiency in two distinct soils. *J. Soil Sci. Plant Nutr.* 17 515–528. 10.4067/S0718-95162017005000037 27315006

[B50] Major J. Rondon M. Molina D. Riha S. J. Lehmann J. (2010). Maize yield and nutrition during 4 years after biochar application to a Colombian savanna oxisol. *Plant Soil* 333 117–128. 10.1007/s11104-010-0327-0

[B51] Malhi S. S. Lemke R. Wang Z. H. Chhabra B. S. (2006). Tillage, nitrogen and crop residue effects on crop yield, nutrient uptake, soil quality, and greenhouse gas emissions. *Soil Tillage Res.* 90 171–183. 10.1016/j.still.2005.09.001

[B52] Maltas A. Kebli H. Oberholzer H. R. Weisskopf P. Sinaj S. (2018). The effects of organic and mineral fertilizers on carbon sequestration, soil properties, and crop yields from a long-term field experiment under a Swiss conventional farming system. *Land Degrad. Dev.* 29 926–938. 10.1002/ldr.2913

[B53] Mazhar S. A. Nawaz M. Khan S. Irshad S. (2018). Impact of urea and farm yard manure on nitrate concentration in soil profile and productivity of wheat crop. *J. Plant Nutr.* 41 2683–2691. 10.1080/01904167.2018.1509994

[B54] Meng L. Ding W. Cai Z. (2005). Long-term application of organic manure and nitrogen fertilizer on N_2_O emissions, soil quality and crop production in a sandy loam soil. *Soil Biol. Biochem.* 37 2037–2045. 10.1016/j.soilbio.2005.03.007

[B55] Mierzwa-Hersztek M. Gondek K. Bajda T. Kopec M. (2019). Use of biochar and a zeolite as adsorbents of mineral pollutions. *Przem. Chem.* 98 1969–1972. 10.15199/62.2019.12.19

[B56] Muhammad B. Adnan M. Munsif F. Fahad S. Saeed M. Wahid F. (2019). Substituting urea by organic wastes for improving maize yield in alkaline soil. *J. Plant Nutr.* 42 2423–2434. 10.1080/01904167.2019.1659344

[B57] Muqaddas B. Ranjha A. M. Abid M. Iqbal M. (2005). Soil physical properties and wheat growth as affected by tillage and farm manure. *Pak. J. Agric. Sci.* 42 56–62.

[B58] Mussarat M. Shair M. Muhammad D. Mian I. A. Khan S. Adnan M. (2021). Accentuating the role of nitrogen to phosphorus ratio on the growth and yield of wheat crop. *Sustainability* 13:2253. 10.3390/su13042253

[B59] Park B. B. Yanai R. D. Sahm J. M. Lee D. K. Abrahamson L. P. (2005). Wood ash effects on plant and soil in a willow bioenergy plantation. *Biomass Bioenergy*. 28 355–365. 10.1016/j.biombioe.2004.09.001

[B60] Peng X. Ye L. L. Wang C. H. Zhou H. Sun B. (2011). Temperature- and duration-dependent rice straw-derived biochar: characteristics and its effects on soil properties of an Ultisol in southern China. *Soil Tillage Res.* 112 159–166. 10.1016/j.still.2011.01.002

[B61] Rawat J. Saxena J. Sanwal P. (2019). “Biochar: a sustainable approach for improving plant growth and soil properties,” in *Biochar-an Imperative Amendment for Soil and the Environment*, eds Abrol V. Sharma P. (London: IntechOpen).

[B62] Reganold J. P. (1995). Soil quality and profitability of biodynamic and conventional farming systems: a review. *Am. J. Altern. Agric.* 10 36–45. 10.1017/S088918930000610X

[B63] Rehman A. Jingdong L. Shahzad B. Chandio A. A. Hussain I. Nabi G. (2015). Economic perspectives of major field crops of Pakistan: an empirical study. *Pac. Sci. Rev. B Humanit. Soc. Sci.* 1 145–158. 10.1016/j.psrb.2016.09.002

[B64] Salem A. K. M. ElKhoby W. M. Abou-Khalifa A. B. Ceesay M. (2011). Effect of nitrogen fertilizer and seedling age on inbred and hybrid rice varieties. *Am. Eurasian J. Agric. Environ. Sci.* 11 640–646.

[B65] Sarma B. Gogoi N. Madhuri B. Priyanka M. (2017). Field evaluation of soil and wheat responses to combined application of hardwood biochar and inorganic fertilizers in acidic sandy loam soil. *Exp. Agric.* 54 507–519. 10.1017/S0014479717000205

[B66] Shah S. A. Shah S. M. Wisal M. Shafi M. Haq N. Samreen S. (2010). Effect of integrated use of organic and inorganic nitrogen sources on wheat yield. *Sarhad J. Agric.* 26 559–563.

[B67] Shah Z. Ahmad M. I. (2006). Effect of integrated use of farm yard manure and urea on yield and nitrogen uptake of wheat. *J. Agric. Biol. Sci.* 1 60–65.

[B68] Shah Z. Shah M. Z. Tariq M. Rahman H. Bakht J. Shafi M. (2012). Survey of citrus orchards for micronutrients deficiency in Swat Valley of north western Pakistan. *Pak. J. Bot.* 44 705–710.

[B69] Shaji H. Chandran V. Mathew L. (2021). “Organic fertilizers as a route to controlled release of nutrients,” in *Controlled Release Fertilizers for Sustainable Agriculture*, eds Lewu F. B. Tatiana V. Sabu T. Rakhimol K. R. (Cambridge, MA: Academic Press), 231–245. 10.1016/b978-0-12-819555-0.00013-3

[B70] Shenbagavalli S. Mahimairaja S. (2012). Characterization and effect of biochar on nitrogen and carbon dynamics in soil. *Int. J. Adv. Biol. Res.* 2 249–255. 10.2134/jeq2011.0133 22751050

[B71] Silva P. S. L. Silva J. D. de Oliveira F. H. T. de Sousa A. K. F. Duda G. P. (2006). Residual effect of cattle manure application on green ear yield and corn grain yield. *Hortic. Bras.* 24 166–169. 10.1590/S0102-05362006000200008

[B72] Singh V. Singh S. P. Singh S. Shivay Y. S. (2013). Growth, yield and nutrient uptake by wheat (*Triticum aestivum* L) as affected by bio-fertilizers, FYM and nitrogen. *Indian J. Agric. Sci.* 83 331–334.

[B73] Steel R. G. D. Torrie J. H. (1980). *Principles and Procedures of Statistical Biometrical Approaches*, 2nd Edn. New York, NY: McGraw Hill Book company.

[B74] Steiner C. Glaser B. Geraldes T. W. Lehmann J. Blum W. E. Zech W. (2008). Nitrogen retention and plant uptake on a highly weathered central Amazonian Ferralsol amended with compost and charcoal. *J. Plant Nutr. Soil Sci.* 171 893–899. 10.1002/jpln.200625199

[B75] Steiner C. Teixeira W. G. Lehmann J. Nehls T. de Macêdo J. L. V. Blum W. E. (2007). Long term effects of manure, charcoal and mineral fertilization on crop production and fertility on a highly weathered Central Amazonian upland soil. *Plant Soil* 291 275–290. 10.1007/s11104-007-9193-9

[B76] Taghizadeh-Toosi A. Clough J. Sherlock R. R. Condron L. M. (2012). Biochar adsorbed ammonia is bioavailable. *Plant Soil* 350 57–69. 10.1007/s11104-011-0870-3

[B77] Torii A. (2012). *Analysis of Field Factors Resulting Frutuations of Yield and Nutritional Uptakes of Forage Rice Leaf Star with Inoculation of an Endophytic Nitrogen Fixing Bacteria TUAT-1*. M.Sc. thesis. Fuchu: Graduate school of agriculture. Tokyo University of Agriculture and Technology.

[B78] Uzoma K. C. Inoue M. Andry H. Fujimaki H. Zahoor A. Nishihara E. (2011). Effect of cow manure biochar on maize productivity under sandy soil condition. *Soil Use Manage.* 27 205–212. 10.1111/j.1475-2743.2011.00340.x

[B79] Van Z. L. Kimber S. Morris S. Chan K. Y. Downie A. Rust J. (2010). Effects of biochar from slow pyrolysis of papermill waste on agronomic performance and soil fertility. *Plant Soil* 327 235–246. 10.1007/s11104-009-0050-x

[B80] Wan Y. Gritsch C. S. Hawkesford M. J. Shewry P. R. (2014). Effects of nitrogen nutrition on the synthesis and deposition of the ω-gliadins of wheat. *Ann. Bot.* 113 607–615. 10.1093/aob/mct291 24344140 PMC3936585

[B81] Wang W. Huang L. Zhu G. Zhang H. Wang Z. Adnan M. (2021). Screening of rice cultivars for nitrogen use efficiency and yield stability under varying nitrogen levels. *J. Plant Growth Regul.* 1–12. 10.1007/s00344-021-10423-1

[B82] Westerman R. L. (1990). *Soil Testing and Plant Analysis. SSSA Book Ser. 3*, 3rd Edn. Madison, WI: SSSA.

[B83] Woldesenbet M. Haileyesus A. (2016). Effect of nitrogen fertilizer on growth, yield and yield components of maize (*Zea mays* L.) in Decha district, Southwestern Ethiopia. *Int. J. Res. Granthaalayah* 4 95–100. 10.29121/granthaalayah.v4.i2.2016.2817

[B84] Yaduvanshi N. P. S. Swarup A. (2005). Effect of continuous use of sodic irrigation water with and without gypsum, farmyard manure, press mud and fertilizer on soil properties and yields of rice and wheat in a long term experiment. *Nutr. Cycl. Agro Ecosyst.* 73 111–118. 10.1007/s10705-005-3361-1

[B85] Zenawi G. Mizan A. (2019). Effect of nitrogen fertilization on the growth and seed yield of sesame (*Sesamum indicum* L.). *Int. J. Agron.* 2019:5027254. 10.1155/2019/5027254

[B86] Zhang A. Bian R. Pan G. Cui L. Hussain Q. Li L. (2012). Effects of biochar amendment on soil quality, crop yield and greenhouse gas emission in a Chinese rice paddy: a field study of 2 consecutive rice growing cycles. *Field Crops Res.* 127 153–160. 10.1016/j.fcr.2011.11.020

[B87] Zhang Y. Li C. Wang Y. Hu Y. Christie P. Zhang J. (2016). Maize yield and soil fertility with combined use of compost and inorganic fertilizers on a calcareous soil on the North China Plain. *Soil Tillage Res.* 155 85–94. 10.1016/j.still.2015.08.006

[B88] Zheng H. Wang Z. Deng X. Herbert S. Xing B. (2013). Impacts of adding biochar on nitrogen retention and bioavailability in agricultural soil. *Geoderma* 206 32–39. 10.1016/j.geoderma.2013.04.018

